# Improving business performance through TPM method: The evidence from the production and processing of crude oil

**DOI:** 10.1371/journal.pone.0274393

**Published:** 2022-09-19

**Authors:** Rafał Drewniak, Zbigniew Drewniak

**Affiliations:** 1 Faculty of Economic Sciences and Management, Nicolaus Copernicus University in Torun, Torun, Poland; 2 Faculty of Management, Bydgoszcz University of Science and Technology, Bydgoszcz, Poland; Al Mansour University College-Baghdad-Iraq, IRAQ

## Abstract

Nowadays, customers expect manufacturers to supply high quality products, on-time deliveries and competitive prices. The consequence of the increased market requirements is the need to maintain high reliability and efficiency of machines and the production process. The existing methods of production management have proved to be inefficient enough to maintain the company’s competitive position on the market. The Total Productive Maintenance (TPM) concept is one of the tools to maximize equipment efficiency by establishing an optimal relationship between people and machines. The aim of the article is to present the issues related to TPM and demonstrate in the process of empirical research that it serves the purpose of improving efficiency and supports quality in the enterprise. The considerations are based on the thesis that TPM is an economical variant of maintenance and guarantees stability, quality and maximization of production efficiency. The article presents the results of empirical research in an enterprise extracting and processing pre-crudes and gas from Caspian Sea. The data from the SAP management system of the investigated enterprise were used. Based on 146 maintenance orders for 40 devices, a correlation between preventive and corrective maintenance was determined using statistical tools. The main goal of the study was to show whether preventive maintenance reduces the occurrence of failures contributing to the elimination of disturbances in the production process. In addition, we analyzed real cases of equipment failures to answer the question whether the procedure of preventive maintenance of equipment in the studied population would prevent the occurrence of these defects. The empirical study demonstrated a clear impact of Preventive Maintenance on limiting the occurrence of equipment failures, and thus production disturbances.

## Introduction

### The essence and importance of TPM—A theoretical background

From the very beginning, the field of quality management (QM) was characterized by practice-based development. Although QM has reached maturity as a field of research based on empirical research, its practitioners still have difficulty adjusting QM implementation to benefit from it [1]. Integration of aspects related to sustainable development and business excellence in the field of product development is considered one of the key objectives of quality management [2]. One of the methods supporting quality management is Total Productive Maintenance (TPM). It is a scientific approach to managing the maintenance of the enterprise, in which each employee is involved in the maintenance, quality and efficiency of the equipment being serviced. TPM is referred to as overall, productivity-oriented maintenance. The concept of TPM was created in Japan as a development of a planned approach to Preventive Maintenance (PM). The main goal of TPM is to reduce the costs of servicing machinery and equipment through more efficient maintenance management and extensive integration of maintenance and production [3, 4]. It is one of the main methods of quality management to improve the production process and is part of the essence of quality assurance that characterizes the "real nature of the object" [5], i.e. product, process, service, etc. TPM comprises the ideas of Preventive Maintenance and Total Quality Management (TQM), which, in combination with employee involvement, is considered a complete management method. In this context, improving productivity through good planning, better processes and full involvement of people should be at the center of attention of quality specialists in the 21st century. Therefore, quality management cannot be rejected as merely an administrative trend, because it provides typical organizational resources on which enterprises can build a sustainable competitive advantage [6]. TPM is considered a new, improved management system that complements the previous TQM concept. Zairi [7] indicates that customer demands and company processes should be matched. Therefore, TQM can provide better planning, better external and internal orientation, better process design, maintenance of the company’s strengths and improvement of weak processes.

The TPM method differs fundamentally from the traditional approach, in that it engages all employees in the management of the maintenance of the enterprise. In fact, quality management requires participation of all employees, which is one of the very important aspects of today’s organization [8]. The distinctive element of TMP is autonomous maintenance, that is activities related to the maintenance of machinery and equipment in the best technical condition and cleanliness, performed by the operators themselves. In this context, Nakajima [9] claims that the analysis of key components of equipment results in reduction of six major sources of production losses: failures, downtime due to adjustments and settings, idle of machines, short technological downtime, quality defects and start-ups of machines and production lines [10].

TPM puts great emphasis on the education of machine operators in order to improve their knowledge of the technology which they are dealing with. Thanks to the training carried out by the maintenance staff and the engineering department, they gain the competence to perform basic maintenance operations, which allows the equipment to be maintained at the optimum level of performance [11]. The technical condition of machines and equipment has a significant impact on production results and is a key aspect in quality management. Machines require human intervention, maintaining cleanliness and improving the efficiency of operations. These conditions may be ensured by promoting among machinery operators a sense of responsibility for the equipment and the workplace. This is one of the main tasks of the TPM philosophy [12]. Ahuja and Singh [13] analyzed the TPM concept based on 8 pillars (Table 1).

**Table 1:** TPM pillars [14].

TPM pillar	Description
Kobetzu Kaizen: elimination of major losses to increase the efficiency of the production system	It is a gradual, timely development and systematic improvement of production, a slow and continuous process improvement in small steps; this means minor improvements resulting from the continuous process of analyzing the existing state of affairs and drawing conclusions (as opposed to the radical innovation path, i.e. drastic change, large investments using new technologies and replacement of equipment). In this sense, Kaizen performs two main functions: maintenance (technology support, maintaining high standards of production management) and process improvement (active participation of employees in the analysis of ongoing processes).
Jishu Hozen	An autonomous maintenance system involving taking over more responsibility and caring for the machine by the operator. It entails the focus of the maintenance department on planned repairs and prevention of failures. Thanks to this approach, technical personnel only deal with machines during periodic and scheduled maintenance, because failures happen very rarely. The main task of Jishu Hozen is to motivate employees to focus daily on maintaining high efficiency of the equipment involved in the production process.
Breakdown maintenance	Implemented by the maintenance department based on the reaction (acting in a given situation, the so-called extinguishing fires) and prevention (actions preventing the occurrence of problems). This means the situation when the machine is operated until it breaks down. This approach to the use of the machine is acceptable only if the failure of the device does not have a major impact on production or does not generate significant costs, with the exception of repair costs.
Preventive Maintenance	It means the daily maintenance of machines based on activities such as cleaning, inspection, lubrication and checking. These activities are aimed at preventing wear of the machines, keeping them in good condition and thus avoiding failures. Periodic checks are aimed at measuring the wear of components and the technical condition.
Education and training	Increasing the skills and competences of operators and maintenance staff to increase their responsibility and independence.
Quality maintenance	Perceived as an element of the chain of operational values, its task is to create added value for the client and ensure the reliability of technical infrastructure; this applies to the maintenance of machines and devices in the best technical condition and cleanliness, carried out by the operators themselves through elimination of wastage, appropriate schedule, upgrading specialists’ qualifications. Its task is to create conditions for production without defects-of-defects or production defects. This goal is achieved by analyzing, identifying and controlling process interactions between people, machines, materials and production methods that could potentially contribute to defects.
TPM in the office	It involves increasing productivity and efficiency in performing administrative functions and support departments (e.g. losses in the course of processes, loss of communication, damage to office equipment).
Management of work safety and environmental protection	Actions aimed at reducing or eliminating the impact of machine operation on the natural environment and ensuring working conditions that do not harm the health and safety of employees.

The holistic approach to effective business management requires taking into account all issues that make up the TPM pillars. It is essential to apply all elements of this method as a holistic management philosophy. The main objectives of TPM are: increasing the Overall Equipment Effectiveness ratio (OEE), increasing the productivity of the crew, achieving a zero defect rate, eliminating production corrections and eliminating total accidents at work [15, 16]. In fact, most improvements use the OEE parameter to determine how successfully TPM has been implemented [17]. The implementation of TPM assumes including people from the maintenance department in the production process, but also increasing the responsibility of machine operators for maintaining the machine park in perfect condition [18]. The key element is the participation of operators in the improvement program and the anticipation and prevention of failures. The cooperation of continuous maintenance employees and operators during the maintenance of repairs contributes to their getting to know each other and increases the skills of operators. It also means a better understanding of the device. TPM is based on preventive anticipation and prevention of defects during machine operation, which allows to extend repair cycles, reduce the number of failures as well as the time of their removal, and better manage spare parts [19]. In fact, preventive maintenance can be performed on a few select items when one fails. One important thing is to apply severity measures that can be used to identify the most important component that requires maintenance [20]. Another important difference between TPM and traditional maintenance is the approach to machine maintenance and inspection. TPM assumes the dominating role of broadly understood prevention, be it in terms of inspections or maintenance, on the production plan [18]. According to the TPM method, the time devoted to modifications and maintenance pays off later, when the machine is still ready for production. The implementation of this objective is possible thanks to the use of tools such as preventive maintenance (prevention of failures), perfect service (modification of equipment to prevent failures and easier handling), prevention of service (design and installation of reliable equipment requiring limited service) and failure handling (repair).

### Benefits of using TPM

During its evolution, TPM has become a comprehensive strategy supporting improved quality in maintenance operations [14, 21]. With maintenance, TPM facilitates plant management, focusing on quality, safety and performance, resulting in a general improvement in organization performance [1, 22]. TPM is the application of TQM principles in maintenance technology [23]; this explains inclusion of TQM principles such as employee engagement, leadership, continuous improvement and customer focus in the TPM model [24, 25]. In contrast, Oakland and Tanner [26] perceive the concept of TQM in the context of its importance for the management of organizations, identifying it with "business excellence", therefore implementation of both TPM and TQM practices contributes in delivering better business performance [27]. Originally, TQM was used in the private business sector [28], which contributed to the wider view of customers as "those for whom we want to create value". The term value refers here to the value chains, not to the financial approach [5]. In order to remain competitive in the market, it is necessary to limit the activities that do not create value [29]. One way to meet this challenge is to implement the TPM concept. When analyzing the benefits of using the TPM method, it should be considered in several areas (Table 2).

**Table 2:** Benefits of using the TPM method [30].

Area	Benefits
Production	Reduction of unplanned downtime through increased availability and machine performance. Effective recognition of hidden, unused production potential. Adaptation of an approach based on the machine’s life cycle to improve the overall efficiency of the equipment. Improvement of the production line capacity due to extensive training of operators.
Quality	Reduction of manufacturing defects and improvement of the stability of the production process.
Costs	Extending the life cycle of machines. Lowering the costs of the maintenance system. Increase in production volume due to improved efficiency and flexibility of the production process. Reduction of losses incurred by downtime and poor quality.
Delivery	Delivery of products at the time required by the customer.
Security	Maintaining a safe working environment; reduction of accidents at work.
Employee morale	Increase in productivity through the high motivation of the staff, achieved by extending the scope of responsibility. Increase in productivity due to a higher level of knowledge, skills and versatility of employees. Improved ability to solve problems. Use of small groups of employees on a voluntary basis to identify the causes of failures, as well as failures and potential modernization of the plant’s equipment.

Among the benefits resulting from the TPM method are direct and indirect. In fact, he application of the discussed method brings significant benefits to both manufacturers and users of technical equipment [31]. According to Hooi and Leong [30], direct profits include: increase in the efficiency of the plant, reduction in the number of returns and complaints, reduction of production costs, increase in customer satisfaction, reduction of accidents at work and compliance with environmental protection principles. Indirect profits, on the other hand, include: increase of self-confidence among the crew, maintenance of good technical condition of workplaces, positive change in the attitude of machine operators, initiation of new concepts in organizational cells, sharing knowledge and experience, and increasing employee responsibility for machines and devices.

### Application of TPM in the crude oil and gas extraction industry

**Fig 1:**
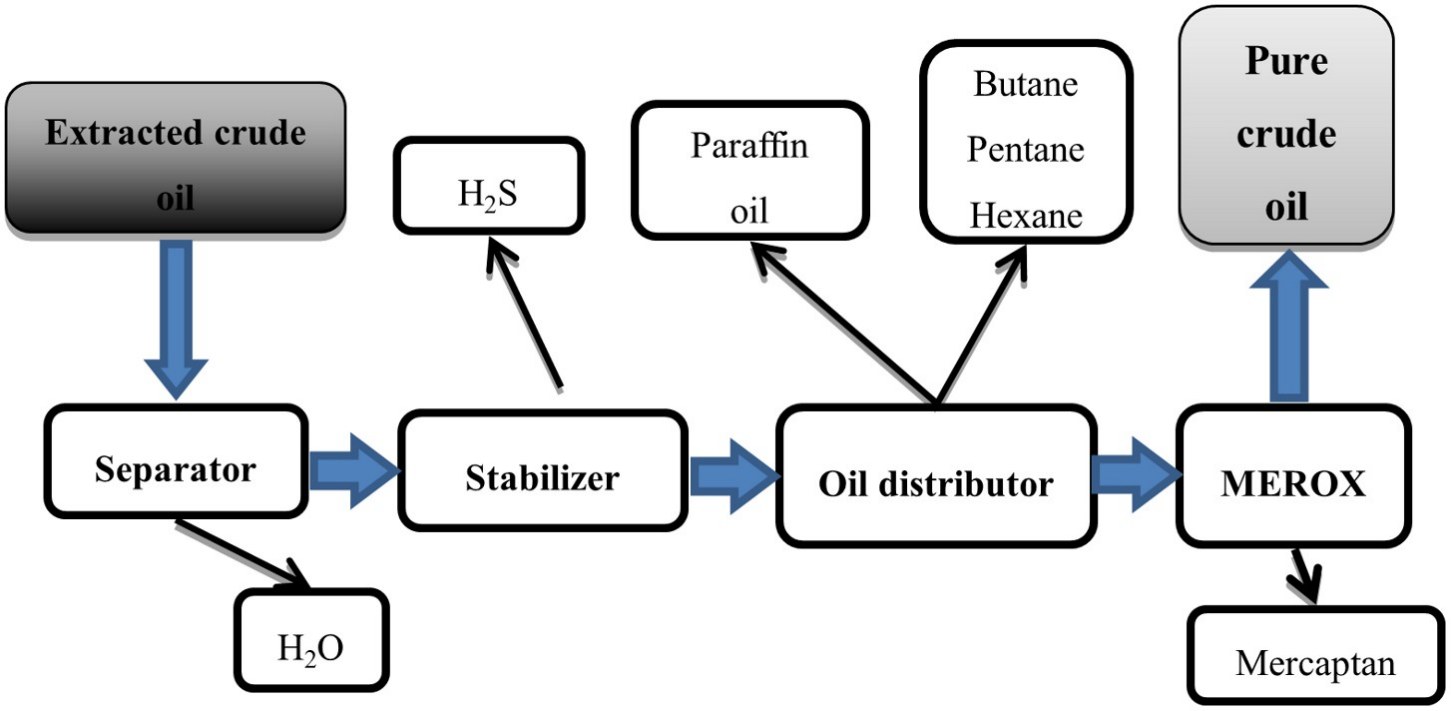
A simplified scheme of the process of crude oil pre-processing.

**Fig 2:**
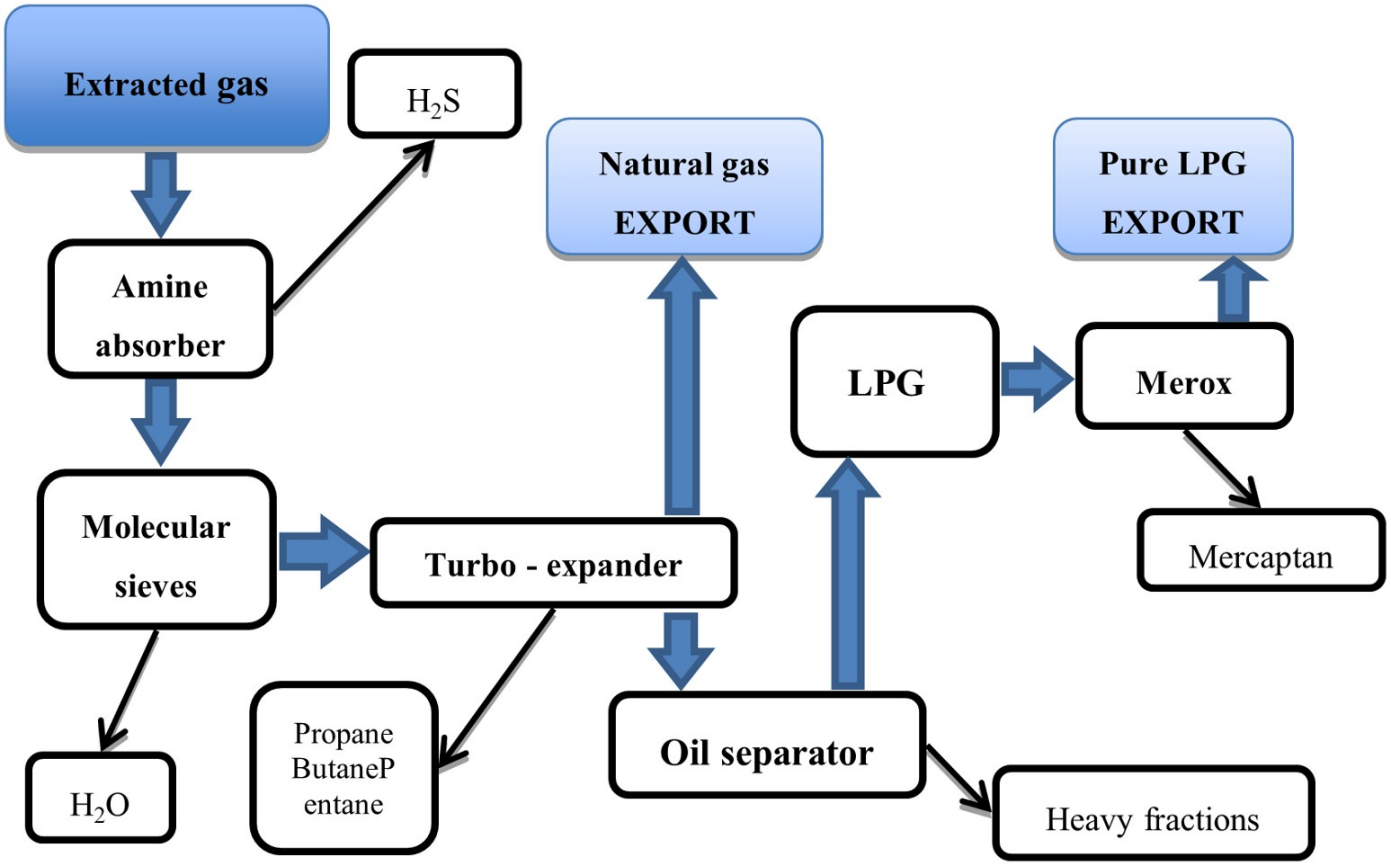
A simplified diagram of the gas processing process.

The article focuses on the analysis of the application of TPM in the crude oil and gas extraction industry. The subject of the analysis is one of the large enterprises operating in Kazakhstan, extracting oil and gas from the bottom of the Caspian Sea, which are then transported by pipelines to a pre-processing plant located on land. The production process consists in removing unwanted components from oil and gas, mainly hydrogen sulfide, mercaptan, light kerosene and water. A simplified process of oil and gas treatment is presented in Figs 1 and 2.

Due to the specificity of production in the tested factory, a disturbance at any stage results in the disruption of the whole process. Excluding any department results in production delays. In order to reduce the probability of such situation, key equipment for production such as, for example, control and protection systems, pumps, power generators, steam boilers or water treatment units, operate in a redundancy system. Despite the large commitment of funds in duplication of critical elements, backup devices constitute only a small part of the entire structure, which remains sensitive to any disruptions.

The oil and gas extracted contain one of the world’s highest contents of deadly toxic and extremely explosive hydrogen sulfide (H2S up to 40% in the oil extracted), making it a special case in terms of health and safety. Great importance in the TPM system used is attached to the human factor. Employees are regularly trained in occupational safety, the use of anti-gas equipment, including the use of personal oxygen masks in emergency situations, fire protection equipment and many other issues related to safety. Training is mandatory and periodically repeated. In addition, weekly discussion meetings on OSH are organized. The company provides employees with all necessary personal protective equipment free of charge. The use of TPM is therefore not only to ensure continuity of production, but also to increase safety.

The research question posed is whether the use of preventive maintenance PM actions reduces the use of corrective maintenance actions. The specific objectives are as follows:

- finding the relationship between the use of PM and CM actions,
- identification of the most common equipment faults and assessment of whether preventive action could prevent a given fault,
- recommendations for improvements.

Our hypothesis is that preventive maintenance PM is an economical variant of maintenance and a guarantee of stability, quality and maximization of production efficiency.

## Methods

### Sampling

In order to carry out the study, service orders from randomly selected devices were analyzed. The surveyed company uses the SAP Blueprint management system, which has a database of maintenance orders. All work orders for level transmitters from the last 3 years have been downloaded from the SAP system. The system collects information on 2578 devices. 40 devices were randomly selected from this group and used for statistical research and analysis. We decided that the group of devices chosen for the study was appropriate because they are directly used in the manufacturing process and thus determine the continuity and quality of the process. The study consisted in analyzing the number and type of work orders for preventive (PM) and corrective actions (CM), and determining the correlation between them. For the studied population of devices, information about 146 orders for preventive and corrective maintenance works was downloaded from the SAS Blueprint system.

### Investigation stages

**Fig 3:**
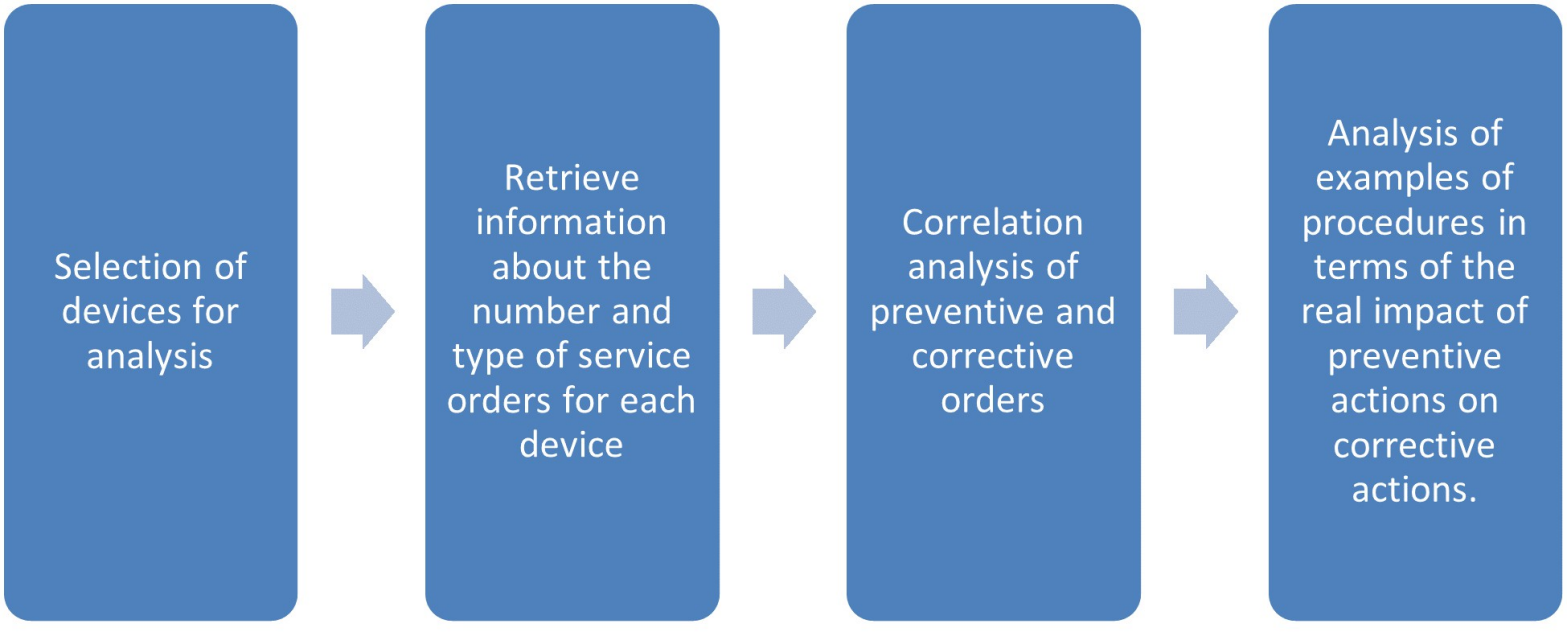
Stages of the study using data from SAP Blueprint.

After extracting all work orders for selected devices, Pearson’s linear correlation coefficient between PM and CM orders was calculated. Based on the result, it was determined whether the number of preventive procedures performed had an impact on the number of device failures. In addition, the preventive and corrective maintenance procedures were analyzed in order to determine whether the PM activities had a real impact on the occurrence of specific CM interventions included in the study group. The impact of the preventive procedure on actual failure prevention was analyzed by contrasting preventive orders with orders for repairs in the tested group of devices. Furthermore, it was possible to evaluate the preventive program in the company and the nature of the most frequent damage to the devices selected for this analysis. The diagram in Fig 3 illustrates the stages of the study.

### Data analysis

Table 3 lists the devices included in the study, together with the number of individual maintenance orders for each device.

**Table 3:** List of the tested device population.

No. of the device	Type of maintenance		No. of the device	Type of maintenance	
	No. of CM	No. of PM		No. of CM	No. of PM
1	0	3	21	4	2
2	0	3	22	1	1
3	0	2	23	3	1
4	0	2	24	3	1
5	0	2	25	4	1
6	1	2	26	3	1
7	2	2	27	3	1
8	4	2	28	4	1
9	5	2	29	3	1
10	0	2	30	4	1
11	0	2	31	3	1
12	1	2	32	5	1
13	0	2	33	3	1
14	0	2	34	3	1
15	0	2	35	3	1
16	2	2	36	4	0
17	0	2	37	6	0
18	0	2	38	5	0
19	0	2	39	5	0
20	0	2	40	4	0

Source: Own study based on data collected from the SAP system of the examined enterprise.

Table 3 presents the number of work orders for each device (level transmitters)—in separate columns there are numbers specifying the amount of corrective maintenances (CM) and preventive maintenances (PM) for each device. Based on the number of orders for maintenance work, the correlation between CM and PM can be determined. Collected data were input into a Pearson correlation to test for relationship among the variables. An alpha value equal to 0.05 was used as a threshold for statistical significance.

The selected group of devices was subject mainly to one type of preventive maintenance order during the period under consideration. It is a wide-ranging test including examination of the general ambient conditions, technical condition of associated devices, mechanical parts, electronics, software and transmitter calibration.

## Results

### PM and CM correlation

**Fig 4:**
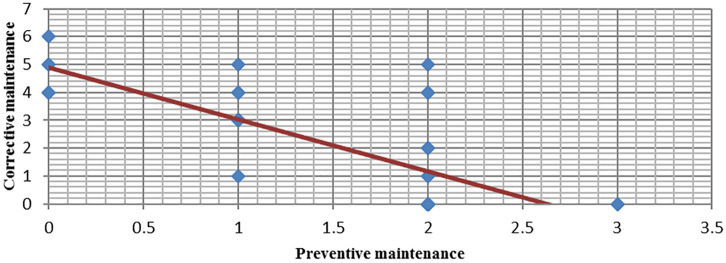
Pearson scattering—CM/PM.

Based on the collected data, the result of the standard deviation σ = 0.77 for PM and σ = 1.91 for CM. Covariance was cov = -1.12. After substituting data from Table 4 and calculations, the obtained Pearson correlation coefficient was -0.756. The value of the coefficient shows a strong negative correlation. It means that as PM maintenance increases, the number of CM maintenance decreases. Fig 4 based on the number of CM and PM maintenances in the test group shows Pearson’s spot spread and illustrates a clear linear downward trend of corrective maintenance as a preventive maintenance function.

**Table 4:** Potential PM impact on CM prevention in the population studied.

No.	Transducer fault reported	SAP damage report	Interpretation of the damage report	The preventive maintenance procedure	Would PM prevent CM?
1	Radar level transmitter spikes–occurrence of short jumps of the output signal not related to real measurement	No damage found	In this case, it means the exchange of electronics. The tests did not locate the fault occurring occasionally and in very short periods of time	No	**NO**: it is not possible to check the transmitter in advance to prevent this type of malfunction
2	Reading abnormal / LT wrong indication–incorrect transmitter measurement	Out of adjustment, calibration drift	Loss of calibration and measurement accuracy	Preventive calibration of the transducer, making corrections and repairs if necessary	**YES**: preventive calibration would prevent loss of measurement accuracy
3	Level Transmitter in fault condition–it is in the "error" state and does not indicate any values	Out of adjustment, calibration drift	Loss of calibration and measurement accuracy	Preventive calibration of the transducer, making corrections and repairs	**YES**: preventive calibration would prevent loss of measurement accuracy
4	LT float stuck–mechanical transducer fault consisting in mechanical blocking of the measuring element	Jammed, sticking, float stacked	Mechanical defect caused by dirt accumulating on the measuring float.	Disassembly of the measuring element for visual inspection and removal of impurities, making repairs	**YES**: The measuring element would be cleaned before complete immobilization
5	Recalibrate level transmitter–was found on the basis of observations of other parallel indicators of transmitter malfunction	Out of adjustment, calibration drift	Loss of calibration and measurement accuracy	Preventive calibration of the transducer, making corrections and possible repairs if necessary	**YES**: Preventive calibration would prevent loss of measurement accuracy
6	Level transmitter got frozen	Frozen, low ambient temperature	The transmitter heating system did not work due to a failure or was not turned on during the winter	Checking of accompanying devices, e.g. the converter’s winter heating system; making any repairs if necessary.	**YES**: A defect in the heating system would be detected before the measuring element was frozen
7	LT reading mismatch–the transducer measurement does not correspond with the measurement of neighboring meters	Out of adjustment, calibration drift	Loss of calibration and measurement accuracy	Preventive calibration of the transducer, making any corrections and repairs	**YES**: Preventive calibration would prevent loss of measurement accuracy
8	Verification–order to verify correct transmitter calibration	Sensor, measuring element worn, fatigue	Natural wear of the measuring element	Preventive calibration of the measuring element, adjustment of the exchange if necessary	**YES**: The item would be replaced before it was completely worn and defective

Source: Own study based on data from the SAP system of the examined enterprise.

Therefore, it can be stated that preventive maintenance in the company clearly limits the occurrence of equipment failures. The high value of the coefficient indicates a vast majority of cases in the population that meet this relationship. The scatterplot (see Fig 4) clearly demonstrates this trend.

### Analysis of the effect of PM on CM prevention

The preventive maintenance procedure includes a wide range of tests, during which a vast majority of the actual and potential technical problems of the device are identified. The previously mentioned defects reported in orders for corrective maintenance, such as incorrect transmitter measurement, mechanical faults, impurities or deviations from the standard of the measuring element, improper functioning of the electronic system, or freezing during winter due to the inoperative heating system, are recognized in the PM process at the initial stage of the defect. Any identified irregularities are removed during the PM process or a corrective adjustment order is initiated with the appropriate priority indicator. This prevents the problem from escalating and reducing the probability of a potential defect that would affect the process or safety in the company.

Table 4 presents descriptions of failure types that occurred in the tested device group, together with the SAP system damage report. The table evaluates the impact of PM maintenance on the potential prevention of failures, and consequently CM maintenance.

As shown in Table 4, only in one case preventive maintenance would not affect the probability of a defect. This applies to the first fault consisting of accidental skip in the transmitter’s output signal. This is a type of damage to the electronic system that cannot be anticipated. In all other cases, PM would prevent the occurrence of failures, because the preventive maintenance procedure involves visual inspection, testing and diagnosis of all listed components whose operation was abnormal or failed. Defects that occurred in the examined group usually present preliminary symptoms, possible to be found before the loss of parameters or failure occurrence.

## Discussion

### Application of TPM in the investigated company

The company providing the data is managed in accordance with all TPM guidelines. Due to the relatively short time that has elapsed since the first launch, the company is still carrying out construction works for additional sub-divisions, which will improve the production conditions. It results from the stage of development of the enterprise is; however, the company’s fundamental philosophy is basically directed towards the Kaizen philosophy, i.e. a slow, gradual improvement of existing processes. In addition, the engineering department collects from the production department information about operational problems on an ongoing basis, and works on providing solutions. It also develops improvements and optimizes production as well as eliminates production limitations, if it does not involve extensive system reconstruction.

The company has a very extensive system of preventive and corrective maintenance. Preventive maintenance is automatically generated by the SAP Blueprint system according to a predetermined plan, based on the recommendations of the equipment manufacturers and technical requirements of the process. The program of preventive maintenance of the elements critical for security and production is particularly well developed. Monitoring of critical security elements such as integrity protection, fire protection and gas hazards is conducted in a separate Facility Safety Report (FSR) system. It guarantees safe maintenance and protection against industrial accidents and disasters. As PM, corrective maintenance is also carried out through the SAP system. After discovering a device fault, the operators enter a repair order into the SAP system. All orders go to the planning department, which makes a 2-week plan for each department. It is adapted to the specificity of production (not all devices are available for the reasons of process limitations), and the efficiency of the department (the number of available specialists and spare parts).

The production processes in the studied company are mainly continuous processes. For this reason, each failure has a direct impact on the size or quality of production. Technical problems often mean disruptions in the oil and gas purification process, which results in lowering the output parameters of the product. If the parameters exceed the limit values, oil and gas must be sent to recycling, which naturally consumes resources and limits production capacity. In worse cases, there may be a disturbance that triggers security systems and directs large amounts of toxic gas to the safety flare, where it is burnt in the atmosphere. This is a big threat to the natural environment. The second negative scenario involves a product that does not meet the requirements of the pipeline, from which it is not possible to withdraw it for reprocessing. In this situation, the product that does not meet the required parameters must reach the consumer. These types of events have a major negative impact on the producer’s reputation. This may result in a temporary suspension of receipt of the export product or termination of the contract. In each case the recipient imposes large fines on the producer.

As already mentioned, failures interfere with the process, which results in production restrictions. Therefore, any disruption entails significant financial losses, which, depending on the type of disruption and production volume, can reach millions of dollars a day. In the continuous processes of oil production it is extremely important to maintain stable process parameters, in particular pressures, levels, temperatures and chemical compositions of intermediate products. There are several thousand measuring and control devices installed in the company. They require constant attention, as well as preventive and corrective maintenance. Level transducers that have been included in the study are examples of devices critical to production and safety. Disturbing of level transducers can result in extremely problematic process situations.

### Potential losses resulting from downtime and damage

In the surveyed company, not all devices were covered by a preventive maintenance plan and there are many that have not yet undergone PM maintenance after the initial commissioning. The relatively short period under study limited to some extent the possibilities of statistical observations, but due to the fact that some devices have not yet undergone any preventive maintenance, the dependence of CM on PM is clearly visible. In the tested range of devices over the past 3 years, 88 failures occurred. Among the tested devices, 57 are transducers of the high priority protection system (Emergency Shutdown System–ESD), the remaining ones are level transmitters critical for the production process. The company also has level transducers installed, which in the event of an alarm or failure cause the entire company to stop, but they are not in the tested group of devices. In the vast majority of cases, the failure of equipment critical for production is associated with a process disruption or complete detention of one of the departments. This in turn results in disrupting production, which always means reduction in the processing and export of crude oil in the range between 10–50 thousand barrels a day (in the surveyed company). Thus, it can be concluded that the failure of a level transmitter in the case of detention of the department incurs production loss of 500–2,000 oil barrels per hour. This means that one-hour demurrage of the department generates losses in the range of USD 52,000–210,000.

As presented in Table 4, most transducers that had regular preventive maintenance did not fail. Typical preventive maintenance is performed by 2 technicians during 8 hours of work. It seems obvious that preventive maintenance costs are quite low considering potential losses. Failure of the main converter (automatic stoppage of the entire production plant), in the case of production at the level of 250,000 barrels a day means a downtime of approximately 570,000 USD per hour. Due to the illustrative nature of the above calculations, the production of natural gas, which takes place parallel to the production of crude oil, is not included here, and the production of gas should also be added to the losses. Starting the plant after an emergency stop lasts–depending on the type of emergency situation–from a few to several hours. Therefore, it can be concluded that the minimal plant downtime losses amount to several million dollars. These calculations are very general and may deviate from reality, but they illustrate the potential consequences of failure of even a single device (depending on its location in the system).

### Recommendations

In production plants where a preventive maintenance program is applied in order to reduce breakdowns and optimize production conditions, the aim is to achieve the target ratio of 70% PM—30% CM. In the surveyed company, we are dealing with the opposite situation (40% PM vs 60% CM). Bearing in mind the results of the study of the relationship between these orders, it should be recommended to use PM activities to a wider extent. The next step in preventive maintenance research in this company should be the analysis of PM and CM relations for other device groups. Should the correlation be similar, it would be practical to launch or improve a preventive maintenance plan for the next tested device group. The development of correct and appropriately adapted preventive maintenance procedures for individual devices is crucial to prevent them from failures. Therefore, one should strive to achieve a relationship in which the amount of preventive maintenances significantly exceeds the number of corrective maintenances, by adapting the prevention program to the specificity of the devices.

## Conclusions

The research and data obtained allow to draw several conclusions. **First of all**, PM is an important element of optimal traffic maintenance. The preventive maintenance plan is one of the pillars of TPM, and preventive maintenance reduces the amount of equipment failures and, consequently, reduces production losses. Conducting periodic scheduled preventive maintenance significantly reduces the breakdown of equipment used for production. It can also be concluded that the cost of conducting regular preventive inspections is insignificantly low in the case of the oil industry, compared to potential losses. It should also be mentioned that any disruption of the production process negatively affects the quality of the output product. Extracted oil and gas must be pre-processed to obtain the appropriate parameters qualifying them for export purposes. Disruptions in production often result in a decrease in the quality of the output product, which, if exported, may not only cause financial losses but also negatively affect the reputation of the company. **Secondly**, preventive maintenance through its positive influence on stabilizing the production process is one of the main ways to maintain productivity and quality of production. A few thousands of control and measurement devices are installed in the analyzed plant, also performing safety functions. The production regime requires equipment reliability, which can only be guaranteed by fully implementing TPM principles, including periodic preventive checks. **Thirdly**, TPM is an economic variant of maintenance and guarantees stability, quality and maximization of production efficiency. When comparing corrective and preventive maintenance procedures, it seems highly probable that most of the reported device failures could have been avoided by conducting regular preventive maintenance. The evidence for this thesis is provided by examples of the tested devices, which, thanks to preventive maintenance, did not undergo any failures. Certainly, the fact that the preventive maintenance plan was not fully implemented adversely affected the size and quality of production in this company. From the beginning, cases of emergency shutdowns of departments or the entire plant occurred, also due to failure of level transmitters.

The presented issues do not cover completely the topic of using TPM in industry, focusing in the research part only on preventive maintenance and its impact on equipment reliability. Achieving high production results is also conditioned by human commitment—from machine operators and maintenance workers to high-level management. Total Productive Maintenance is one of the Lean Manufacturing methods. One of its main goals is to maintain production equipment in good technical condition through preventive maintenance of machinery and equipment used on a scheduled basis and comprehensively in the enterprise. This is done by providing an extensive technical equipment service system that covers the entire life of the machine. The main goal is to achieve zero failure rate of equipment involved in the production process. It is the maximization of efficiency by increasing the time of availability of machines for product manufacturing–in particular devices of critical importance for production. Present very dynamic and rapidly changing environment, as well as global competition between organizations have led to higher requirements for production organizations. As for the contribution of TPM to production efficiency, Miyake and Enkawa (21) investigated the use of JIT, TQC and TPM paradigms to improve the efficiency of production systems. They emphasize that TPM stands out as a vehicle that is very conducive to the implementation of improvements (from elementary remedies to technologically complex projects), with the participation of technical staff, foremen and employees [32].

The aim of the article was to demonstrate the relationship between preventive and corrective actions and thus justify the use and further expansion of the TPM concept. According to the published literature, there aren’t many TPM implementation methods accessible for the manufacturing industry. An examination of the effective application of TPM in is actually required to fulfilled this gap. The finding of the literature indicates that there is a need of TPM model specially developed for the manufacturing sector [33]. In further work on the analysis of the phenomenon in the mining sector, it would be reasonable to use tools to measure the effectiveness of TPM. One such indicator is Overall Equipment Effectiveness (OEE) widely used as an important quantitative indicator for measuring performance in manufacturing operations [34]. The use of OEE varies from industry to industry and is tailored to the specific requirements of the industry.

TPM is a complex process and can only be implemented through a comprehensive and systematic implementation in the production unit. It engages all employees of the company. One of the major differences between TPM and other maintenance concepts is the involvement of machine operators in corrective and preventive actions. They must be properly targeted and trained accordingly. Expanding the knowledge and skills of employees is one of the pillars of this system. The classic separation between maintenance staff and operators in the case of TPM is very limited. The engineering and planning department must also be involved in creating its architecture. They must create technical procedures, inspection and repair plans for the equipment to be adapted to the specificity of the plant and production type.
